# eIF2A, an initiator tRNA carrier refractory to eIF2α kinases, functions synergistically with eIF5B

**DOI:** 10.1007/s00018-018-2870-4

**Published:** 2018-07-17

**Authors:** Eunah Kim, Joon Hyun Kim, Keunhee Seo, Ka Young Hong, Seon Woo A. An, Junyoung Kwon, Seung-Jae V. Lee, Sung Key Jang

**Affiliations:** 1 PBC, Department of Life Sciences, Pohang University of Science and Technology, Cheongam-ro 77, Nam-gu, Pohang-si, Gyeongsangbuk-do 37673 Republic of Korea; 2 School of Interdisciplinary Bioscience and Bioengineering, Pohang University of Science and Technology, Cheongam-ro 77, Nam-gu, Pohang-si, Gyeongsangbuk-do 37673 Republic of Korea

**Keywords:** Translation initiation, eIF2A, eIF5B, Evolution of initiator tRNA carriers

## Abstract

The initiator tRNA (Met-tRNA_i_^Met^) at the P site of the small ribosomal subunit plays an important role in the recognition of an mRNA start codon. In bacteria, the initiator tRNA carrier, IF2, facilitates the positioning of Met-tRNA_i_^Met^ on the small ribosomal subunit. Eukarya contain the Met-tRNA_i_^Met^ carrier, eIF2 (unrelated to IF2), whose carrier activity is inhibited under stress conditions by the phosphorylation of its α-subunit by stress-activated eIF2α kinases. The stress-resistant initiator tRNA carrier, eIF2A, was recently uncovered and shown to load Met-tRNA_i_^Met^ on the 40S ribosomal subunit associated with a stress-resistant mRNA under stress conditions. Here, we report that eIF2A interacts and functionally cooperates with eIF5B (a homolog of IF2), and we describe the functional domains of eIF2A that are required for its binding of Met-tRNA_i_^Met^, eIF5B, and a stress-resistant mRNA. The results indicate that the eukaryotic eIF5B–eIF2A complex functionally mimics the bacterial IF2 containing ribosome-, GTP-, and initiator tRNA-binding domains in a single polypeptide.

**Electronic supplementary material:**

The online version of this article (10.1007/s00018-018-2870-4) contains supplementary material, which is available to authorized users.

## Introduction

The translation of an mRNA begins when a small ribosomal subunit finds the initiation codon of an mRNA. The presence of an initiator tRNA (Met-tRNA_i_^Met^) at the P site of the small ribosomal subunit (the 30S and 40S ribosomes of prokaryotes and eukaryotes, respectively) critically enables the recognition of the initiation codon [1]. Translation factors called initiator tRNA carriers facilitate the placement of Met-tRNA_i_^Met^ onto the P site of small ribosomal subunits. The bacterial translation factor, IF2, is a typical initiator tRNA carrier that facilitates the allocation of a formylmethionine-charged initiator tRNA (fMet-tRNA_i_^fMet^) onto the P site of the 30S ribosomal subunit. To ensure correct placement, IF2: (1) associates with fMet-tRNA_i_^fMet^ via an interaction between the C-terminal region of IF2 and the formyl group of fMet-tRNA_i_^fMet^ [2]; (2) interacts with the 30S ribosome through the N-terminal region of IF2 [3]; (3) facilitates the joining of the large ribosomal subunit (the 50S ribosome) with the 30S ribosome [4]; and concomitantly (4) hydrolyzes GTP and dissociates from the 30S ribosome [5, 6].

Eukaryotic IF2 (eIF2) is the major initiator tRNA carrier responsible for loading eukaryotic Met-tRNA_i_^Met^ onto the 40S ribosomal subunit. eIF2 is composed of three polypeptides, the α, β, and γ subunits. Of them, the γ subunit has GTPase and Met-tRNA_i_^Met^-binding activities [7]; the β subunit facilitates the association of eIF2 and the 40S ribosome through an interaction with eIF3 [8]; and the α subunit regulates the activity of eIF2 [9]. More specifically, the phosphorylation of a regulatory serine (serine 51) of the eIF2α subunit (in yeast) inhibits the Met-tRNA_i_^Met^ carrier activity of eIF2 via the sequestration of eIF2B, which is the guanosine nucleotide exchange factor responsible for the GDP–GTP exchange activity of eIF2 [10, 11]. A number of eIF2α kinases are activated by various stress signals, including oxidative stress [heme-regulated inhibitor (HRI) or EIF2AK1], viral infection [protein kinase double-stranded RNA-dependent (PKR) or EIF2AK2], ER overload [PKR-like ER kinase (PERK) or EIF2AK3], and ROS accumulation or amino acid starvation [general control non-derepressible-2 (GCN2) or EIF2AK4]. Under the relevant stress conditions, these kinases phosphorylate the regulatory serine of eIF2α, thereby repressing the translation of most eukaryotic mRNAs [12]. After eIF2 delivers Met-tRNA_i_^Met^, the carrier is released from the 40S ribosome through a conformational change induced by GTP hydrolysis [13]. eIF5B, a homolog of IF2, stabilizes the association of initiator tRNA with the 40S ribosome and helps to ensure the correct positioning of Met-tRNA_i_^Met^ on the P site of the 40S ribosome when the start codon of an mRNA pairs with the anticodon of Met-tRNA_i_^Met^ [14, 15].

The translations of several mRNAs are known to be refractory to the translational inhibition mediated by the phosphorylation of eIF2α [16]. Numerous studies have sought to understand the molecular basis of the persistent translation of specific mRNAs under stress conditions. Many such studies have focused on identifying the carrier(s) for Met-tRNA_i_^Met^ in the translation of the hepatitis C viral (HCV) mRNA, which is efficiently translated under stress conditions when the eIF2α subunit is heavily phosphorylated [17]. Several cellular proteins have been suggested to participate in recruiting Met-tRNA_i_^Met^ to a 40S ribosome associated with an HCV mRNA containing an internal ribosome entry site (IRES) [18–21]. In addition, eIF2A, which stimulates the GTP-independent binding of Met-tRNA_i_^Met^ to a 40S ribosome programmed with AUG [22, 23], was reported to be responsible for the persistent translation of HCV mRNA under stress conditions [24]. The authors showed that siRNA-mediated knockdown of eIF2A severely repressed HCV mRNA translation under stress conditions. Moreover, the addition of recombinant eIF2A proteins to an eIF2A-depleted in vitro translation system restored HCV mRNA translation in the presence of phosphorylated eIF2α. eIF2A strongly interacts with Met-tRNA_i_^Met^ and with stem-loop IIId of the HCV IRES, which was shown to be essential for HCV mRNA translation under stress conditions [24]. The data thus indicate that eIF2A is responsible for supplying Met-tRNA_i_^Met^ to a 40S ribosome associated with an HCV mRNA, and that the direct interaction of eIF2A with the HCV mRNA is required for its selective translation under stress conditions. Several papers, which suggest that eIF2A functions in translation initiation, have been published after the report. For instance, eIF2A was shown to mediate the stress-resistant translation of c-Src mRNA, which is essential for cell proliferation under stress conditions [25]. The authors demonstrated that eIF2A facilitates tRNA_i_^Met^ loading onto the 40S ribosome in a c-Src mRNA-dependent manner. Similarly, to the direct interaction of eIF2A with the HCV mRNA, eIF2A directly interacts with c-Src mRNA [25]. The results indicate that the direct interaction of eIF2A with a specific mRNA plays an important role in determining the mRNA specificity of Met-tRNA_i_^Met^ loading to a 40S ribosome. Interestingly, recent reports suggest that eIF2A participates in translation of mRNAs utilizing CUG codon as a translation start codon [26–29]. The reports suggested that eIF2A participates in antigen presentation by MHC class 1 molecule, stress responses, and tumor initiation. However, the molecular basis of the activities of eIF2A in translation of these mRNAs has been poorly understood. Curiously, an eIF2A-null mouse has been generated recently, and the eIF2A-null mouse did not show an apparent abnormality under normal growth conditions [30]. It would be interesting if the eIF2A-null mouse shows an abnormality under stress conditions.

A comparison of eIF2A with IF2 reveals functional similarities and differences. Both proteins directly bind to initiator tRNAs and deliver them to small ribosomal subunits in an AUG-dependent manner [31], thereby differing from eIF2, which delivers Met-tRNA_i_^Met^ to the 40S ribosome in an AUG-independent manner [23]. Unlike IF2, however, eIF2A neither directly interacts with the small subunit of ribosome nor has the GTPase activity that is required for IF2 and eIF2 to dissociate from the ribosome after they deliver Met-tRNA_i_^Met^. The mechanisms how eIF2A deploys Met-tRNA_i_^Met^ in the P site of the 40S ribosomal subunit and how eIF2A dissociates from the 40S ribosome after delivering Met-tRNA_i_^Met^ remains to be elucidated. We thus hypothesized that it may work cooperatively with another, GTPase activity-bearing protein, to function as a Met-tRNA_i_^Met^ carrier.

Here, we set out to identify a protein that functions cooperatively with eIF2A as a Met-tRNA_i_^Met^ carrier. We focused on a translation initiation factor eIF5B that was shown to be genetically related to yeast eIF2A. A previous study showed that knockout of both eIF2A and eIF5B yields a synthetically sick phenotype in the yeast *Saccharomyces cerevisiae*, suggesting that these proteins function in the same pathway [23]. As eIF5B has ribosome-binding and GTPase activities that could potentially complement the lack of such activities in eIF2A, we herein investigated the potential cooperative function of these proteins to act as a Met-tRNA_i_^Met^ carrier.

We first confirmed that eIF2A and eIF5B show a genetic interaction in animals, using *Caenorhabditis elegans* (*C. elegans*) as a multicellular model organism. In the eIF2A-null mutant, eIF5B knockdown triggered a severe delay in development, suggesting that eIF5B and eIF2A function in the same pathway of *C. elegans* (Fig. S1). To further examine the molecular basis for this genetic effect, we investigated the potential physical interaction of these proteins, and identified an interaction between purified eIF2A and eIF5B proteins. We determined the domains in eIF2A required for its interactions with eIF5B, Met-tRNA_i_^Met^, and mRNA. Furthermore, we performed experiments, showing that eIF5B augments the activity of eIF2A in loading Met-tRNA_i_^Met^ onto a 40S ribosome associated with an HCV mRNA. Finally, we analyzed the functional domains of eIF2A associated with eIF5B with respect to those of well-known bacterial fMet-tRNA_i_^fMet^ carrier IF2 as follows: in bacteria, IF2 itself exhibits initiator tRNA-binding, ribosome-binding, GTPase, and large subunit-joining activities; and in Eukarya, the eIF5B–eIF2A complex possesses the equivalent activities of IF2. In addition, the eIF5B–eIF2A complex has a specific mRNA-binding activity that does not exist in IF2. The results provide insight into the molecular basis of how the eIF5B–eIF2A complex enables the translation of specific mRNAs to occur under stress conditions.

## Materials and methods

### Cell culture, transfection, and immunoprecipitation

293FT cells were cultivated in Dulbecco’s modified Eagle’s medium (Gibco BRL) supplemented with 10% fetal bovine serum (Clontech). Plasmids were transfected to cells using Lipofectamine (Invitrogen). The transfected cells were cultivated on plates, washed with cold PBS (pH 7.4), and then lysed in 500 μl of ice-cold buffer A [0.1% NP-40, 40 mM HEPES–KOH (pH 7.5), 100 mM KCl, 1 mM EDTA, 10 mM β-glycerophosphate, 10 mM NaF, 2 mM Na_3_VO_4_, and 1 mM PMSF]. The solution was sonicated on ice and centrifuged to yield whole-cell extracts (WCEs). For immunoprecipitation, WCEs were incubated with 10 μl of anti-Flag M2 affinity gel (Flag resin; Sigma) at 4 °C for 2 h with constant rotation. The beads were collected and washed four times with the same buffer, and the bead–bound proteins were resolved by SDS-PAGE and analyzed by western blotting.

### Purification of recombinant proteins and GST pull-down assay

His-tagged eIF2A was expressed in *E. coli* strain M15 using plasmid pQE31/His–eIF2A, and the expressed protein was purified as previously described [24]. GST-fused eIF5B and GST-fused eIF2A variants were expressed in *E. coli* strain Bl21. The GST-fused proteins were purified and GST pull-down assays were performed, both as previously described [32]. The purified proteins were visualized by coomassie blue staining (Fig. S2).

### In vitro transcription and pull down with biotinylated RNAs

For the production of biotinylated tRNAs and HCV IRESs, plasmids pRL-CMV/Met-tRNA_i_^Met^ and pRL-CMV/tRNA^Leu^, which were previously reported [24], were treated by *BstN*1, whereas pRL-CMV/HCV IRES was treated by *Acc*I [24]. The linearized DNAs were used as templates for in vitro transcription reactions with T7 RNA polymerase in the presence of 1 mM biotinylated UTP. RNA pull-down experiments were performed using purified GST–eIF2A (1 μg) or eIF2A-overexpressing 293FT cell lysates (3 mg) and biotinylated RNAs (3 μg), as previously described [24].

### Filter-binding assays

40S ribosomes were prepared from HeLa cells as described previously [32]. The amounts of components are as follows: 2 pmol of [^32^P]tRNA_i_^Met^ (10,000 c.p.m./pmol), 2.5 pmol of 40S ribosomal subunit, 2 pmol of HCV IRES, and 3 pmol each of eIF2A and eIF5B. The procedures were performed as described previously [24].

### Sucrose density gradient analysis

Polysome profile analyses were performed with the cell extracts of mock- or tunicamycin (2 μg/ml)-treated 293FT cells. Briefly, the cells were treated with cycloheximide (100 μg/ml) for 30 min on ice, and lysed with 1 ml of lysis buffer [300 mM KOAc, 10 mM MgCl_2_, 50 mM HEPES–KOH (pH 7.5), 100 mM NH_4_Cl, 5 mM DTT, 0.1 mM EDTA, 0.5% NP-40, and 100 μg/ml cycloheximide]. The sucrose gradients were prepared with polysome buffer [300 mM KOAc, 10 mM MgCl_2_, 50 mM HEPES–KOH (pH 7.5), 100 mM NH_4_Cl, 2 mM DTT, and 0.1 mM EDTA]. The cell lysate (500 μg) was resolved on 5–45% sucrose gradients through ultracentrifugation for 3 h at 38,000 rpm in an SW41Ti rotor (Beckman). We collected 0.25 ml fractions via a gradient density fractionator (Brandel) using upward displacement with 60% (w/v) sucrose at a flow rate of 0.25 ml/min. Continuous monitoring was performed at an absorbance of 254 nm using an Econo UV monitor (Bio-Rad). Proteins in every second fraction were methanol-precipitated and analyzed by western blotting. rRNAs in the fractions were analyzed by agarose gel electrophoresis after phenol/chloroform extraction followed by ethanol precipitation, and rRNAs were visualized by ethidium bromide staining.

## Results

### Genetic interaction between eIF2A and eIF5B

The presence of a genetic interaction between eIF2A and eIF5B was previously uncovered in a yeast knockout study [23], where the single knockouts of eIF2A and eIF5B yielded mild and moderate slow-growth phenotypes, respectively, whereas double knockout of eIF2A and eIF5B yielded a very severe slow-growth phenotype. The “synthetically sick” phenotype of the double mutant was taken as suggesting that these proteins function in the same biological pathway. In the present study, we first set out to confirm the relationship of eIF2A and eIF5B in animals, using knockdown and knockout experiments in the model organism, *C. elegans*. Briefly, we characterized a null mutant worm lacking *eIF2A* (E04D5.1) and examined the effect of *eIF5B*/*iffb*-*1* knockdown (Fig. S1). More specifically, we monitored the effect of *eIF5B* knockdown on wild-type (panels 1 and 2 in Fig. S1a) and *eIF2A*-null worms (panels 3 and 4 in Fig. S1a) at 52 h after hatching. Knockout of *eIF2A* yielded a very mild slow-development phenotype, as shown in Fig. S1b (compare columns 1 and 3). Knockdown of *eIF5B* in the wild-type background yielded a slow-development phenotype (compare panel 1 with 2 in Fig. S1a; compare column 1 with 2 in Fig. S1b). Importantly, knockdown of *eIF5B* in the *eIF2A* mutant background yielded a very severe slow-development phenotype (Fig. S1a and S1b), suggesting that eIF5B and eIF2A work together in the same pathway.

### Physical interaction between eIF2A and eIF5B

**Fig. 1:**
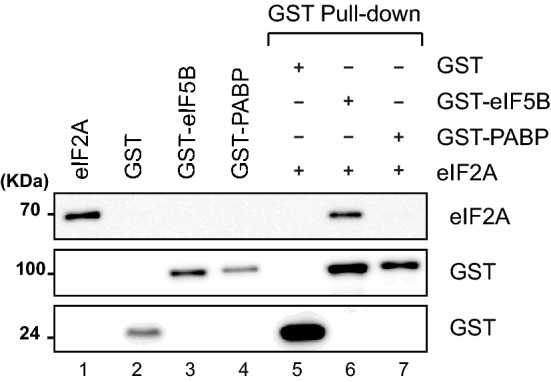
eIF2A directly interacts with eIF5B. GST pull-down experiments were performed with recombinant His–eIF2A, GST–eIF5B (587–1220), GST–PABP, and GST, which were purified from *E. coli* extracts. The GST pull-down samples (lanes 5–7) were resolved by SDS-PAGE and visualized by western blotting with anti-His (R&D Systems) for His-tagged eIF2A or anti-GST [32] for GST-fusion proteins. The input proteins (lanes 1–3) correspond to 10% of the proteins used for immunoprecipitation

To gain insight into the molecular basis underlying the genetic interaction between eIF2A and eIF5B, we investigated their potential direct interaction using glutathione *S*-transferase (GST) pull-down assays with purified recombinant proteins. A GST-fused ΔeIF5B_587–1220_ protein (containing GST and residues 587–1220 of eIF5B; designated GST–eIF5B) was expressed in *E. coli* and purified. This recombinant protein was chosen, because the N-terminal region of eIF5B is not essential for its function, and ΔeIF5B_587–1220_ was previously shown to be fully functional in vivo and in vitro [33]. Similarly, a His-tagged eIF2A protein was expressed in *E. coli* and purified. Our results revealed that eIF2A co-precipitated with GST–eIF5B, but not with negative control GST and GST–PABP proteins (Fig. 1, compare lanes 5, 6, and 7). This indicates that eIF2A directly interacts with eIF5B (Fig. 1).

### The middle domain of eIF2A (residues 462–501) is necessary and sufficient for the interaction with eIF5B

**Fig. 2:**
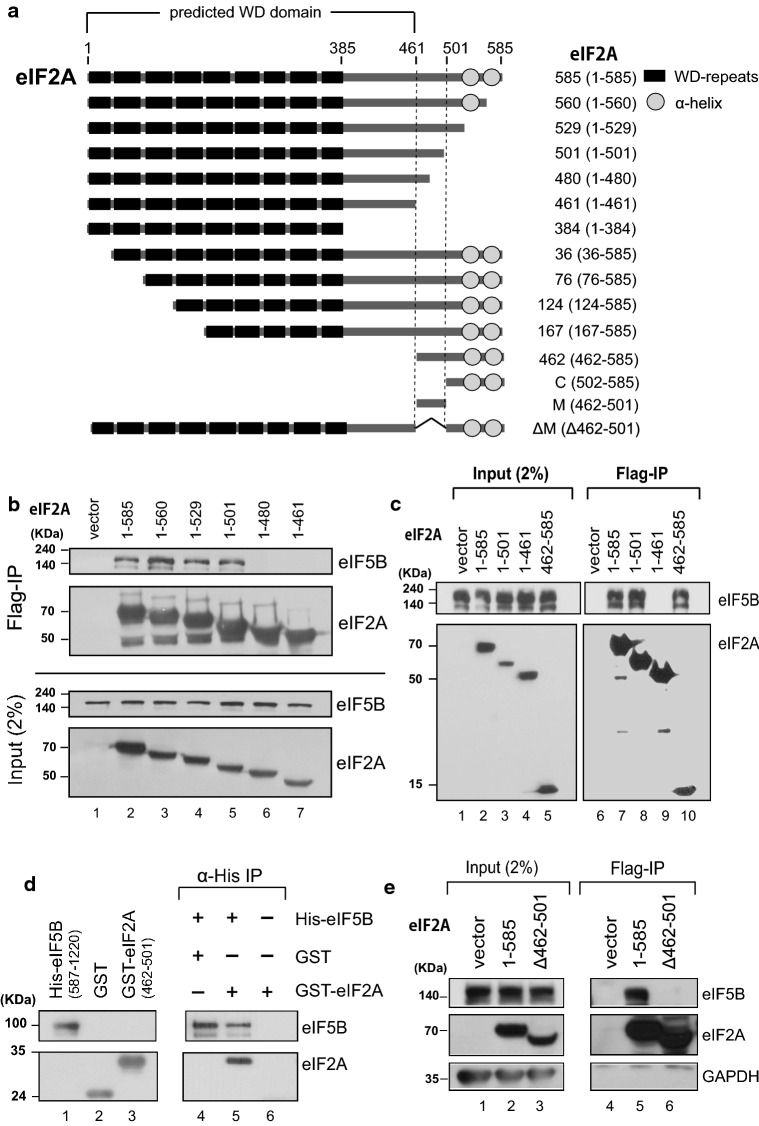
Determining the eIF2A domain required for the interaction with eIF5B. **a** Schematic diagram of eIF2A and its derivatives. **b**, **c** Immunoprecipitation was performed using lysates of 293FT cells expressing Flag-tagged eIF2A derivatives. Flag-tagged eIF2A proteins were precipitated with Flag resin, and the endogenous eIF5B proteins associated with the eIF2A derivatives were visualized by western blotting with anti-eIF5B (ProteinTech Group), anti-Flag (Sigma), and anti-GAPDH (AbD Serotec). **d** Co-precipitation of purified eIF2A and eIF5B. His-tagged eIF5B (587–1220) and GST-fused eIF2A (462–501) polypeptides were mixed, and the former was precipitated with an anti-His-conjugated resin. Co-precipitated GST–eIF2A (462–501), which corresponds to the M domain, was visualized by western blotting with anti-GST. **e** Co-precipitation experiments were performed as described in panels (**b**, **c**), except that we used a plasmid encoding Flag-eIF2A (Δ462–501)

To determine the region of eIF2A that is required for its interaction with eIF5B, we performed co-immunoprecipitation (Co-IP) experiments with various deletion mutants of eIF2A. We generated mammalian expression vectors encoding various parts of eIF2A, which were chosen based on their size and the predicted structures of eIF2A (Fig. 2a). To predict the functional domains of eIF2A, we analyzed the conservation of amino acid sequences from yeast to human (Fig. S3). We also predicted the three-dimensional structure of the WD domain of human eIF2A based on the crystal structure of this domain of eIF2A in the fission yeast, *Schizosaccharomyces pombe* (Fig. S4) [34]. The eIF2A of *S. pombe* was shown to have nine-bladed β-propeller fold which is unconventional as a WD domain. The C-terminal end of eIF2A is predicted to contain two alpha helices, as assessed using the Phyre2 program (Fig. S4b) [35]. Interactions between endogenous eIF5B and ectopically expressed Flag-tagged variants of eIF2A were observed by Co-IP experiments (Fig. 2b, c). As expected based on the co-precipitation of the purified proteins, endogenous eIF5B co-precipitated with full-length eIF2A (residues 1–585). eIF5B interacted with eIF2A (1–501) but not with eIF2A (1–480) (Fig. 2b), indicating that the C-terminal border of the eIF5B-interacting domain of eIF2A was located between residues 480 and 501. eIF2A (462–585) interacted with eIF5B (lane 10 in Fig. 2c), indicating that the WD domain of eIF2A is not required for the interaction. To confirm that the middle part of eIF2A (residues 462–501; designated as ‘M domain’) is sufficient for the interaction with eIF5B, we synthesized and purified GST–eIF2A (462–501). Indeed, purified eIF5B co-precipitated with GST–eIF2A (462–501), indicating that the M domain of eIF2A is sufficient for its interaction with eIF5B (Fig. 2d). To test whether the M domain of eIF2A is absolutely required for the interaction with eIF5B, we generated a deletion mutant lacking the M domain: eIF2A (Δ462–501). This protein failed to interact with eIF5B in a co-precipitation experiment (Fig. 2e). Taken together, our co-precipitation data indicate that the M domain of eIF2A is necessary and sufficient for its interaction with eIF5B.

To determine the eIF2A-binding domain in eIF5B, GST pull-down experiments were performed with purified recombinant proteins (Fig. S4c). eIF5B is composed of four functional domains (domains G to IV) [36]. The domains G and II are responsible for binding to the 40S ribosomal subunit, and the domain IV is connected with domain III via a long α-helix (H12 of 40 Å). The pull-down experiments indicated that the domains G + II + III + IV of eIF5B strongly interacts with eIF2A, and the domain IV and domains III + IV weakly bind to eIF2A (lanes 9, 11, and 12 in Fig. S4c). However, either domain III or domains II + III did not bind to eIF2A (lanes 10 and 13 in Fig. S4c). The results indicate that the domain IV of eIF5B is the minimal domain for the interaction with eIF2A and that the domains G to IV are needed for the full capacity of interaction with eIF2A.

### The WD domain of eIF2A interacts with initiator tRNA

**Fig. 3:**
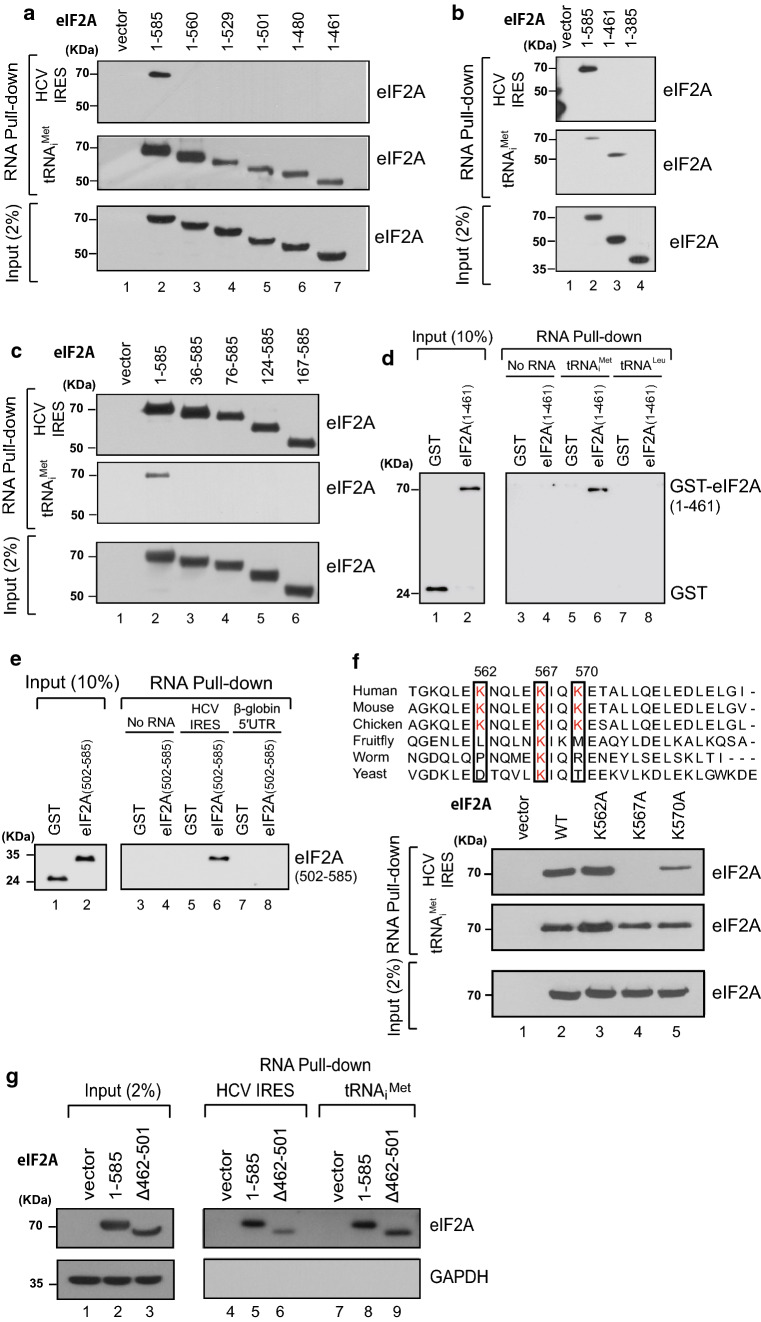
Determining the eIF2A domain required for binding to tRNA_i_^Met^ and the HCV mRNA. **a**–**c** RNA pull-down experiments were performed with biotinylated tRNA_i_^Met^ and a biotinylated HCV RNA corresponding to the IRES element (HCV IRES), using lysates of 293FT cells transfected with plasmids expressing various Flag-tagged deletion mutants of eIF2A. The RNA-bound proteins were precipitated with streptavidin-conjugated agarose resin and visualized by western blotting with anti-Flag (Sigma). **d** RNA pull-down experiments were performed with biotinylated RNAs (tRNA_i_^Met^ and tRNA^Leu^) and purified recombinant protein GST-fused eIF2A (1–461), which corresponds to the WD domain. **e** RNA pull-down experiments were performed with biotinylated RNAs (HCV IRES and β-globin 5′UTR) and purified GST–eIF2A (502–585), which corresponds to the C domain. **f** RNA pull-down experiments were performed with biotinylated tRNA_i_^Met^ and biotinylated HCV IRES using lysates of 293FT cells expressing various substitution mutants of Flag-tagged eIF2A. **g** RNA pull-down experiments were performed with biotinylated tRNA_i_^Met^ and biotinylated HCV IRES using lysates of 293FT cells expressing Flag-eIF2A (Δ462–501) with an internal deletion of the M domain. The amounts of input lysates and proteins loaded to control lanes are indicated in each panel

In a previous report [24], we showed that eIF2A interacts with both Met-tRNA_i_^Met^ and the IRES elements of a target HCV mRNA. In this study, we set out to determine the domains of eIF2A that are required for its interactions with the HCV IRES and Met-tRNA_i_^Met^. We performed RNA pull-down experiments using extracts from 293FT cells ectopically expressing various deletion mutants of eIF2A (see Fig. 2a) and biotinylated RNAs corresponding to the HCV IRES or tRNA_i_^Met^. Our results revealed that the very C-terminal region (residues 561–585) of eIF2A is essential for the interaction with the HCV IRES but not tRNA_i_^Met^ (Fig. 3a). Conversely, the very N-terminal-end region (residues 1–35) was essential for the interaction with tRNA_i_^Met^ but not the HCV IRES (Fig. 3c). Biotinylated tRNA_i_^Met^ interacted with eIF2A (1–461) but not eIF2A (1–385) (Fig. 3b), indicating that the C-terminal border for the interaction with tRNA_i_^Met^ is located between residues 386 and 461 of eIF2A. We further confirmed the interaction between eIF2A (1–461) and tRNA_i_^Met^ using purified GST-fused eIF2A (1–461) (Fig. 3d). These results indicate that the WD domain of eIF2A directly interacts with tRNA_i_^Met^, whereas the alpha helix-forming C-terminal part of eIF2A (residues 502–585; designated as C domain), which is preceded by the M domain, is sufficient for interaction with the HCV IRES (Fig. 3e). Given our finding that certain lysine residues of the C domain are conserved (Fig. 3f), we examined the potential involvement of these residues in the interaction with the HCV IRES. We generated eIF2A proteins with lysine-to-alanine mutations, and tested their ability to bind the HCV mRNA. Mutation of lysine 567, which is the most highly conserved lysine in this region, specifically abrogated the binding of eIF2A with the HCV IRES (Fig. 3f; Fig. S5a), indicating that lysine 567 of eIF2A plays a key role in the interaction with the HCV IRES. We further confirmed the roles of the WD and C domains in the interactions with the tRNA_i_^Met^ and HCV IRES, respectively, using an internal deletion mutant of eIF2A lacking residues 462–501 [i.e., the M domain; eIF2A (Δ462–501)]. Consistent with our above-described findings, this mutant eIF2A maintained its ability to bind both tRNA_i_^Met^ and the HCV IRES (Fig. 3g). Moreover, we showed that tRNA_i_^Met^ and the HCV IRES do not compete for binding to eIF2A (Fig. S5b). Together, these data indicate that eIF2A is composed of at least three separable functional domains, WD, M, and C, which are required for the abilities of eIF2A to interact with Met-tRNA_i_^Met^, eIF5B, and a target mRNA, respectively (Fig. 6a).

### eIF5B synergistically augments the ability of eIF2A to load Met-tRNA_i_^Met^ onto the 40S ribosomal subunit

**Fig. 4:**
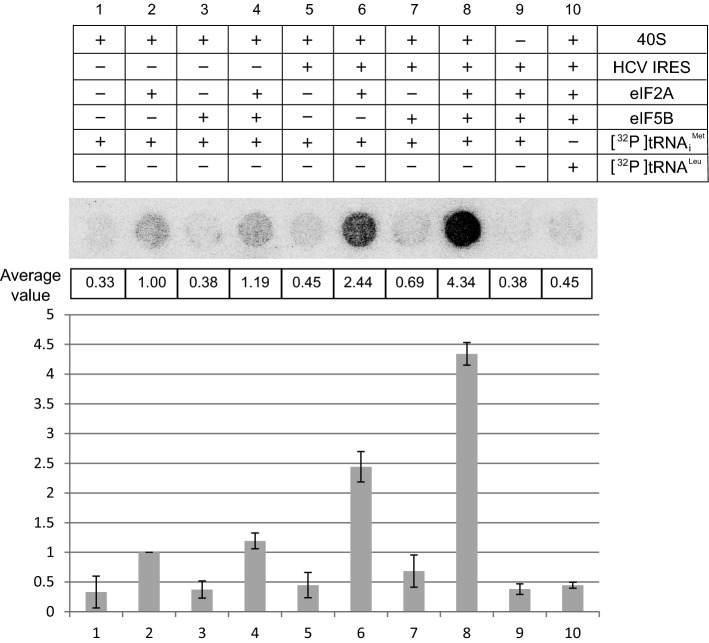
eIF2A and eIF5B synergistically facilitate the loading of tRNA_i_^Met^ onto the 40S ribosomal subunit. The loading of [^32^P]tRNA_i_^Met^ onto the 40S ribosomal subunit was monitored by a filter-binding assay [24] performed using radiolabeled tRNAs, 40S ribosomes, and various combinations of eIF2A, eIF5B, and the HCV IRES, as depicted in the upper panel. Experiments were performed three times, and average values are presented in the middle panel, along with a representative autoradiogram. The columns and bars in the bottom panel represent the means and ± standard deviations, respectively

It was previously reported that eIF5B and eIF2A independently facilitate the loading of Met-tRNA_i_^Met^ onto the 40S ribosome in an HCV mRNA-dependent manner [18, 19, 24]. Here, we confirmed the tRNA_i_^Met^-loading activities of eIF2A and eIF5B using in vitro filter-binding assays with [^32^P]-labeled tRNA_i_^Met^ and purified 40S ribosomes (Fig. 4). Since our above-described results showed that these proteins interact with each other, we further examined tRNA_i_^Met^ loading in the presence of both eIF2A and eIF5B. Consistent with the previous report [24], eIF2A increased the loading of tRNA_i_^Met^ to the 40S ribosome by threefold in the absence of the HCV IRES (compare columns 2 with 1 in Fig. 4), and the presence of the HCV IRES further increased tRNA_i_^Met^ loading by an additional 2.5-fold (compare column 6 with 2 in Fig. 4, and column 5 with 2 in Fig. S6a). eIF5B showed a little (if any) tRNA_i_^Met^-loading activity in the absence of the HCV IRES (compare column 3 with 1 in Fig. 4). Consistent with the previous report, we observed a significant induction (about twofold) of eIF5B-mediated tRNA_i_^Met^ loading in the presence of the HCV mRNA (compare column 7 with columns 1 and 3 in Fig. 4, and column 4 with 1 in Fig. S6a). However, the overall tRNA_i_^Met^-loading capability of eIF5B was about fourfold weaker than that of eIF2A (compare columns 6 with 7 in Fig. 4, and column 5 with 4 in Fig. S6a). This may suggest that eIF2A plays the major role in loading Met-tRNA_i_^Met^ onto an HCV mRNA-associated 40S ribosome. Interestingly, the eIF2A-mediated tRNA_i_^Met^ loading of the 40S ribosome was further enhanced by about twofold in the presence of eIF5B (compare columns 8 with 6 in Fig. 4). Under the same experimental conditions, a negative control tRNA^Leu^ was not loaded to 40S ribosome (column 10 in Fig. 4). Taken together, the data indicate that eIF2A can specifically load Met-tRNA_i_^Met^ onto the 40S ribosome associated with an HCV mRNA, and that eIF5B synergistically augments this loading of Met-tRNA_i_^Met^ onto the 40S ribosomal subunit.

### Both eIF2A and eIF5B are associated with the 40S ribosomal subunit under stress conditions

**Fig. 5:**
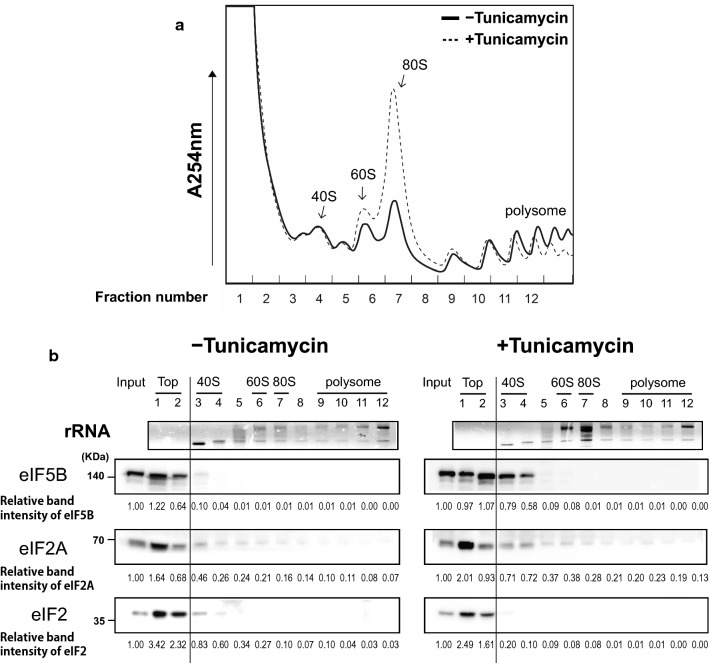
Both eIF2A and eIF5B are associated with 40S ribosome under stress conditions. Sucrose gradient analyses were performed with cell extracts from mock- or tunicamycin (2 μg/ml)-treated 293FT cells. Cell extracts were centrifuged on 5–45% sucrose gradients. **a** Ribosomal distributions were monitored by measuring absorbance at 254 nm. **b** Fractions of the sucrose gradients were collected, and every second fraction was used for the analyses of rRNAs and proteins. The RNAs in each selected fraction were resolved by agarose gel electrophoresis after phenol/chloroform extraction followed by ethanol precipitation, and rRNAs were visualized by ethidium bromide staining (panel rRNA). Proteins in each selected fraction were methanol-precipitated and analyzed by western blotting with antibodies against eIF5B (ProteinTech Group), eIF2A (ProteinTech Group), and the α subunit of eIF2 (Novus) (panels eIF5B, eIF2A and eIF2, respectively). The protein band intensities were quantified, and the relative values of the band intensity with respect to the same protein in the 10% of cell extract (Input) (which was set to 1) are depicted as numbers

The previous studies showed that eIF5B, which directly associates with the 40S ribosomal subunit, is released from the ribosome via a conformational change that is triggered by the hydrolysis of GTP on eIF5B [18, 33], but the mechanism of association and dissociation of eIF2A with the ribosome is unknown. Given our findings that eIF2A and eIF5B directly interact and synergistically facilitate the loading of Met-tRNA_i_^Met^ onto the 40S ribosome, we speculated that eIF2A may associate and dissociate with the 40S ribosome through a direct interaction with eIF5B. To test this possibility, we used western blot analyses to detect eIF5B, eIF2A, and eIF2α in fractions obtained from sucrose gradient analyses performed with lysates of cells cultivated under normal (without tunicamycin) and stress (tunicamycin-treated) conditions (Fig. 5). As expected, tunicamycin treatment induced phosphorylation of eIF2α (Fig. S7) and inhibited overall translation as indicated by the decrease of polysomal fractions (dotted line in Fig. 5a). Under normal conditions, both eIF2A and eIF5B were mainly found in the top fraction (Fig. 5b). This may suggest that these proteins do not participate in translation under normal conditions, or that the sucrose gradient analyses caused them to dissociate from ribosomal complexes (perhaps, because their ribosomal binding affinities are weak under normal conditions). Interestingly, we observed increases of both eIF5B and eIF2A in the 40S fractions of stressed (tunicamycin-treated) cell lysates (fractions 3 and 4 in Fig. 5b). These results strongly suggest that both eIF2A and eIF5B participate in translation under stress conditions, and that they may act together. Further investigation is needed to clearly prove that eIF2A and eIF5B associate and dissociate with the 40S ribosome in a complex. Curiously, we observed some eIF2A proteins in the polysome fractions under both normal and stress conditions. We speculate that these eIF2A proteins may be directly associated with various mRNAs through the mRNA-binding domain (C domain) of eIF2A, independent of eIF5B or any ribosome.

## Discussion

**Fig. 6:**
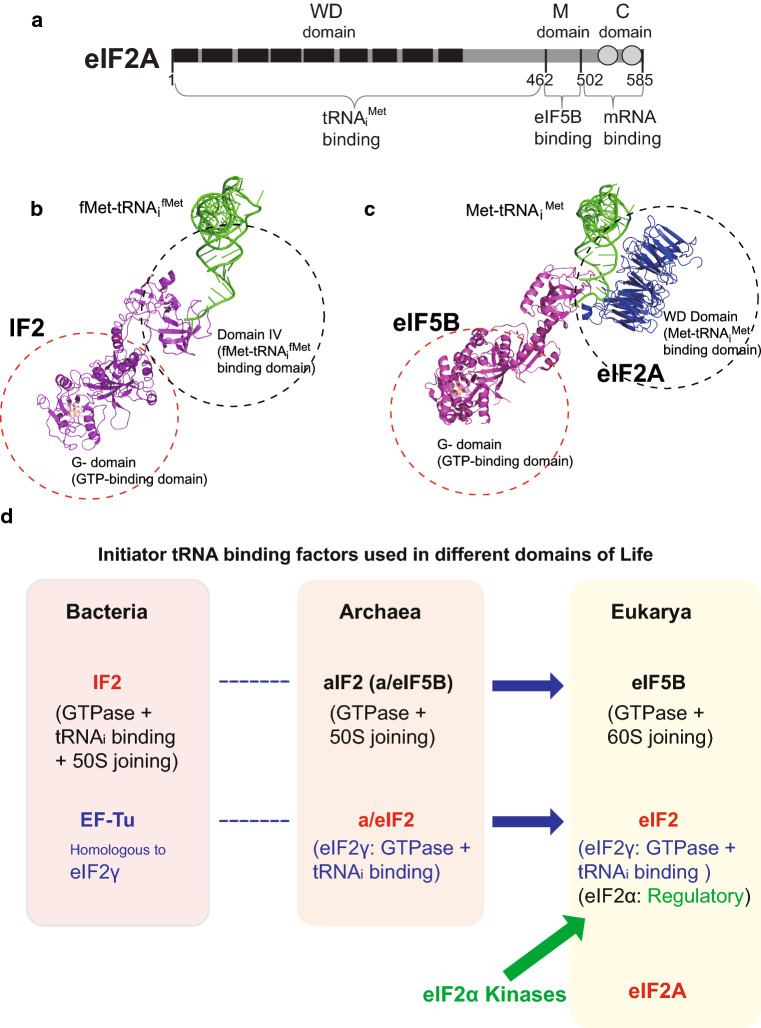
Comparison of initiator tRNA carriers. **a** Structural and functional domains in eIF2A. WD-repeat sequences and α-helical regions are depicted as closed boxes and open circles, respectively. **b** Ribbon diagram showing IF2 (magenta) complexed with fMet-tRNA_i_^Met^, as previously reported by Allen et al. (PDB: 1ZO1) [59]. **c** Model of the eIF5B/eIF2A/Met-tRNA_i_^Met^ complex. The structure of eIF2A (blue) was predicted using the Phyre2 server [60] and visualized with the PyMOL program [35]. The structures of eIF5B (magenta) and Met-tRNA_i_^Met^ were previously reported by Fernandez et al. (PDB: 4BYX) [14]. **d** Evolutionary perspective on the initiator tRNA carriers. The initiator tRNA carrier homologs and their functions are depicted

eIF2A, which was recently shown to act as an initiator tRNA carrier, functions under stress conditions when the activity of eIF2, the major carrier of Met-tRNA_i_^Met^, was compromised by various stress-related kinases [24]. Although eIF2A was shown to interact with both Met-tRNA_i_^Met^ and a specific mRNA (HCV mRNA), the domains responsible for these interactions remained unknown. Moreover, eIF2A does not possess a GTPase domain, which would be expected to trigger the conformational change required to liberate the protein from the 40S ribosome. Thus, the mechanism responsible for releasing eIF2A from the 40S ribosome after delivering Met-tRNA_i_^Met^ was also unknown. We speculated that this necessary GTPase function could be provided by a partner protein that cooperates with eIF2A to initiate translation. As yeast eIF5B is genetically related to eIF2A [23], we herein set out to investigate the potential of eIF5B to function as a counterpart of eIF2A in the delivery of Met-tRNA_i_^Met^. We first confirmed the genetic interaction between eIF2A and eIF5B in a model animal (*C. elegans*). The *C. elegans* homolog of eIF5B, *iffb*-*1*, is known to be required for larval development, germ cell proliferation, and differentiation [37]. We used an *eIF2A* mutant worm and an RNAi against *eIF5B* to investigate whether E04D5.1/*eIF2A* and *iffb*-*1*/*eIF5B* are required during development. As previously reported, *eIF5B*-knockdown worms showed a slow-development phenotype that was enhanced by knockout of *eIF2A* (Fig. S1). These data demonstrate that the genetic interaction of eIF2A and eIF5B previously shown in yeast also exists in animals. We next studied the molecular basis of this genetic interaction, and found that eIF2A physically interacts with eIF5B (Fig. 1), and that this interaction occurs through the M domain of eIF2A (residues 462–501) (Figs. 2, 6a). Moreover, we found that the WD (residues 1–461) and C (residues 502–585) domains of eIF2A are responsible for its interactions with tRNA_i_^Met^ and mRNA, respectively (Figs. 3, 6a). Lysine 567 of eIF2A, which is phylogenetically well conserved, turned out to be essential for mRNA binding (Fig. 3; Fig. S5a). In addition, we showed that the lysine 567 of eIF2A is essential for mRNA-dependent loading of tRNA_i_^Met^ onto the 40S ribosomal subunit using in vitro filter-binding assay (Fig. S8). The functional domains of eIF2A identified in this study are depicted in Fig. 6a.

Comparison of the structural and functional domains of the bacterial fMet-tRNA_i_^fMet^ carrier, IF2 (Fig. 6b), with those of eIF2A and eIF5B allowed us to predict the relative positions of eIF2A, eIF5B, and Met-tRNA_i_^Met^ during their interaction (Fig. 6c). In addition, we predicted the eIF2A structure complexed with the 40S ribosomal subunit associated with eIF5B, tRNA_i_^Met^, and HCV IRES based on our results (Fig. S4b). The N-terminal part of eIF2A, which is composed of a WD domain associated with Met-tRNA_i_^Met^, may localize over the P site of 40S ribosome to load Met-tRNA_i_^Met^ on the P site. The structure of M domain of eIF2A could not be predicted by an available structure prediction program, but we could speculate the position of M domain through the interactions between eIF2A and eIF5B and between eIF2A and HCV IRES. The domain IV of eIF5B is likely to stretch out over the P site of 40S ribosomal subunit and interact with the M domain of eIF2A (depicted as a dotted line in Fig. S4b). The M domain of eIF2A is likely to position over the P and E sites of 40S ribosomal subunit and is connected to the C domain that is predicted to be composed of two consecutive alpha helices that binds to the IIId domain of HCV IRES (Fig. S4b). According to the cryo-EM structure of HCV IRES associated with the 40S ribosomal subunit, the IIId domain of HCV IRES sticks out over the platform of 40S ribosome [38]. It is plausible that the C-terminal end of C domain of eIF2A touches the extended part of IIId domain where the 40S ribosomal subunit is not associated. With these configurations of 40S ribosome, eIF5B, eIF2A, Met-tRNA_i_^Met^, and HCV IRES, all of the components may form a complex through RNA–protein–protein–RNA interactions (Fig. S4b). The interaction between eIF2A and a specific mRNA seems to play a key role in selecting mRNAs refractory to stress-dependent translational repression [24]. Recently, we reported that the stress-resistant translation of c-Src mRNA directed by an IRES element, which is essential for cell proliferation under stress conditions, is also mediated by eIF2A [25]. A direct interaction between eIF2A and c-Src IRES was shown to be essential for eIF2A-mediated translation [25]. Interestingly, both HCV and c-Src IRESs, which are known to use eIF2A as a Met-tRNA_i_^Met^ carrier, interact with 40S ribosome directly. However, it is not clear whether only the IRES-containing mRNAs utilize eIF2A as a Met-tRNA_i_^Met^ carrier. Our current investigations on the mRNAs associated with eIF2A under stress conditions with a genome-wide approach revealed that many cellular mRNAs associate with eIF2A under stress conditions (data not shown). The functionalities of the massive interactions between eIF2A and various mRNAs are under investigation.

Our study of the potential functional cooperation between eIF2A and eIF5B revealed that eIF5B synergistically augments the eIF2A-mediated loading of Met-tRNA_i_^Met^ onto the 40S ribosome (Fig. 4). Interestingly, the start codon is critical for the HCV IRES-dependent loading of Met-tRNA_i_^Met^ onto the 40S ribosome, which is mediated by eIF2A. A mutant IRES, which contains a substitution mutation of the start codon from AUG to AAA, failed to augment eIF2A-mediated loading of tRNA_i_^Met^ onto the 40S ribosome, even though the mRNA-independent loading of tRNA_i_^Met^ remained in the presence of the mutant HCV IRES (Fig. S6). On the contrary, the eIF5B-mediated loading of tRNA_i_^Met^ onto the 40S ribosome was completely abolished when the mutant HCV IRES was used in the filter-binding assay (Fig. S6b). In addition, the synergistic activation of tRNA_i_^Met^ loading onto the 40S ribosome by eIF2A and eIF5B (Fig. 4) was abolished when the mutant HCV IRES was used in the filter-binding assay (Fig. S6b). The difference of tRNA_i_^Met^-loading capability of these proteins is likely attributed to the binding affinity of these proteins with tRNA_i_^Met^. That is, eIF2A but not eIF5B strongly interacts with tRNA_i_^Met^. This indicates that the codon–anticodon interaction between the HCV IRES and the Met-tRNA_i_^Met^ plays an important role in mRNA-dependent loading of Met-tRNA_i_^Met^ onto the 40S ribosome, which is mediated by either eIF2A or eIF5B. Based on these results and the previous reports, we speculate that eIF5B enables eIF2A to bind ribosomes, while eIF2A enables eIF5B to bind Met-tRNA_i_^Met^ associated with an mRNA through a codon–anticodon interaction.

Finally, we used sucrose density gradient analyses to examine the dispersion patterns of eIF2A and eIF5B proteins under normal and stress conditions. Under normal conditions, both proteins were found mostly in the top and 40S fractions (fractions 1 and 2 in Fig. 5). The distribution pattern of eIF5B is somewhat surprising, as it has been suggested to promote the correct placement of Met-tRNA_i_^Met^ [39], stabilize Met-tRNA_i_^Met^ at the P site [40], and facilitate the association of the 60S and 40S ribosomes [33]. Nevertheless, our findings are consistent with the distribution pattern of eIF5B reported previously [41]. It is noteworthy that knockout of eIF5B yields a slow-growth phenotype, suggesting that its activity is not essential for yeast growth under normal conditions [23]. As an alternative explanation for the observed distribution of eIF5B in the sucrose density gradient, we speculate that eIF5B might have been dissociated from the 40S ribosome during the analytic process. This possibility should be re-investigated in the future. Critically, under stress conditions, eIF5B and eIF2A showed much more co-elution with the 40S ribosome (e.g., they were both found in fraction 3 and 4 in tunicamycin-treated cells) (Fig. 5b). Moreover, our results indicate that these proteins are released from the 40S ribosome later than eIF2 under stress conditions (fraction 3 in Fig. 5b). The distribution patterns of eIF2A and eIF5B in the sucrose density gradient analyses were similar under normal and stress conditions. This may indicate that these two proteins are released from the 40S ribosome as a complex, before the joining of the 60S ribosomal subunit, perhaps, via a conformational change of eIF5B induced by its hydrolysis of GTP. However, further investigation is needed to clearly prove that these two proteins associate and dissociate with the 40S ribosome as a complex. Notably, low levels of eIF2A were detected in all tested fractions (Fig. 5b). It is likely to reflect the direct (ribosome- and eIF5B-independent) association of the C domain of eIF2A with mRNAs.

### Evolutionary perspective on initiator tRNA carriers (Fig. 6d)

Given what our present results indicate regarding the tight communication between eIF5B and eIF2A, it seems worthwhile to re-examine the general features and relationships among the carriers of Met-tRNA_i_^Met^. In the case of bacteria, a single translation factor, IF2, facilitates the loading of fMet-tRNA_i_^fMet^ on the P site of the small ribosomal subunit. The N-terminal part of IF2 is responsible for interacting with the 30S ribosome and binding GTP, while the C-terminal part, which is connected to the N-terminal domain via a long α-helix, interacts with a formylmethionine-charged initiator tRNA (fMet-tRNA_i_^fMet^) via the formyl group [42]. In addition, IF2 facilitates the joining of the large ribosomal subunit (the 50S ribosome) to the translational pre-initiation complex composed of the 30S ribosome, mRNA, and fMet-tRNA_i_^fMet^ [43–45]. The hydrolysis of GTP on IF2, which is activated by the joining of the large ribosomal subunit, occurs concomitantly with the dissociation of IF2 from the 70S ribosomal complex. The hydrolysis of GTP is believed to induce conformational changes in IF2, triggering this dissociation [6]. Archaea express a homolog of IF2 called archaeal IF2 (aIF2, also known as a/eIF5B), which contains GTP- and ribosome-binding domains [46]. Unlike bacterial IF2, however, aIF2 may not interact with Met-tRNA_i_^Met^ strongly, since the formylation of methionine, which is essential for the interaction with IF2, does not occur in Archaea and Eukarya [47]. Therefore, aIF2 alone may not be able to recruit Met-tRNA_i_^Met^ to the P site of the 30S ribosome. Instead, Archaea express an alternative Met-tRNA_i_^Met^ carrier called a/eIF2, the homolog of eukaryotic translation initiation factor eIF2, which recruits Met-tRNA_i_^Met^ to the small ribosomal subunit [48]. The cooperative action of a/eIF5B and a/eIF2 facilitates the loading of Met-tRNA_i_^Met^ at the P site of the small ribosomal subunit [39, 46]. The α subunit of a/eIF2 does not undergo kinase-mediated phosphorylation in Archaea; this mode of repression exists only in Eukarya. Indeed, the Eukarya express structural and functional homologs of archaeal a/eIF2 and a/eIF5B, called eIF2 and eIF5B, respectively. eIF5B is a ribosome-dependent GTPase that mediates ribosomal subunit joining [1]. Similar to a/eIF5B, eIF5B contains a G domain and domain II, which confer GTPase and ribosome-binding activities, respectively [36]. In addition, similar to a/eIF5B, eIF5B binds weakly to Met-tRNA_i_^Met^ [46], which makes it less likely to function as an initiator tRNA carrier by itself. Instead, the concerted actions of eIF5B and the Met-tRNA_i_^Met^ carrier, eIF2, facilitate the loading of Met-tRNA_i_^Met^ to the P site of the 40S ribosome and stabilize the ribosome-tRNA_i_^Met^ complex. Moreover, eIF5B accelerates the joining of the ribosomal subunits [49].

In Eukarya, an additional Met-tRNA_i_^Met^ carrier, eIF2A, exists from yeast to human. It functions when the activity of eIF2 is compromised by phosphorylation of its α subunit under stress conditions. We propose that eIF2A coevolved with the eIF2α kinases for the translation of specific mRNAs that must be translated even when the function of eIF2 is compromised by stress responses. Various sets of eIF2α kinases exist in different groups of Eukarya (Table 1). Among the eIF2α kinases, only GCN2 (EIF2KA4), which is activated by ROS and amino acid starvation, exist in most Eukarya (from yeast to human, Table 1). This may suggest that the basic needs of translational regulation by eIF2α kinase are related to the functions of GCN2. Considering the functions of GCN2 and their difference between prokaryotes and eukaryotes, we speculate that the absolute necessity of translational regulation by eIF2α kinases is to protect cells from ROS generated by dysfunctional mitochondria. Moreover, GCN2 may also play an important role in maintaining mitochondrial function and promoting cell growth during mitochondrial stress as reported previously [50]. Notably, the eukaryotes that lack mitochondria, such as the Diplomonads and Trichomonas, do not possess homologs of either an eIF2α kinase or eIF2A. This strongly suggests that the GCN2 have evolved primarily to deal with ROS generated by dysfunctional mitochondria and/or to protect mitochondria. Other eIF2α kinases may have evolved to cope with stresses other than ROS accumulation. eIF2A is, therefore, likely to have evolved to facilitate the translation of specific mRNAs that are required to maintain cellular functions under stress conditions, including the accumulation of ROS.

**Table 1:** eIF2α kinases of various eukaryotic organisms

Kinase	Yeast	*C. elegans*	Mammal	Stresses
HRI (EIF2AK1)	**○**		**○**	Heme levels, oxidative stress [51, 52]
PKR (EIF2AK2)			**○**	ds RNA [53]
PERK (EIF2AK3)		**○**	**○**	ER stress, hypoxia [54, 55]
GCN2 (EIF2AK4)	**○**	**○**	**○**	Amino acid starvation, UV irradiation, ROS [56–58]

The eIF2α kinases and their activating stresses are depicted for yeasts, *C. elegans*, and mammals

### Electronic supplementary material

Below is the link to the electronic supplementary material.

**Fig. S1** Genetic interaction between *eIF2A*/E04D5.1 and *eIF5B*/*iffb*-*1* of *C. elegans.* **a** Development was monitored among wild-type and *eIF2A*/E04D5.1 mutant animals treated with control RNAi or *eIF5B*/*iffb*-*1* RNAi. Worms were maintained and cultured at 20˚C. Wild-type N2 (Bristol) and E04D5.1/*eIF2A* mutants were allowed to lay eggs on control RNAi or *iffb*-*1*/*eIF5B* RNAi plates for 12 h. Approximately 50 eggs were transferred to new control RNAi or *iffb*-*1*/*eIF5B* RNAi plates, and the eggs were allowed to grow until wild-type animals developed to adult stages. Worms of each group were assessed for their developmental stages at 52 h post-hatching, and pictures were taken. The scale bars represent 1 mm. **b** Graphs depict the proportions of worms at the developmental stages of young adult and adult at 52 h post-hatching. *** P < 0.001. P values were derived by Chi square test.

**Fig. S2** Visualization of purified recombinant proteins. Recombinant proteins used in binding experiment were resolved by SDS-PAGE and visualized by Coomassie blue staining. The first lanes are the protein-prestained protein markers (NEB). Arrowheads depict the proteins of interest.

**Fig. S3** Sequence alignment of eIF2A homologs from various organisms. Alignment was performed with CLUSTALW and the figure was prepared using ESPript [61].

**Fig. S4** Predicted structure of eIF2A on 40S ribosome complex. **a** Functional domains of eIF2A. The N-terminal, middle, and C-terminal domains of eIF2A participate in bindings to Met-tRNA_i_^Met^, eIF5B, and mRNA, respectively. **b** Predicted eIF2A structure complexed with the 40S ribosomal subunit that is associated with eIF5B, Met-tRNA_i_^Met^, and HCV IRES (PDB ID: 4UPY, 4UQ5). The structure of eIF2A was predicted by the Phyre2 server [60] and visualized with the PyMOL program [35]. The structure of M domain was not able to predict. The dashed line illustrates the M domain that touches the domain IV of eIF5B and is connected to the C domain. **c** Determination of the eIF2A-binding site in eIF5B. The His–eIF2A protein (2 nM) was incubated with GST–eIF5B derivatives (2 nM each) at 4 ˚C for 1 h in 1 ml of binding buffer [0.2% NP-40, 40 mM HEPES–KOH (pH 7.5), 100 mM KCl, 1 mM EDTA, 10 mM β-glycerophosphate, 10 mM NaF, 2 mM Na_3_VO_4_, 2.5% BSA, and 1 mM PMSF], and then, 10 μl of Glutathione Separose 4B resin (GE healthcare) was added to the mixture. The mixture was further incubated at 4˚C for 20 min with a Rotamix. The beads were washed four times with 1 ml of binding buffer. The input samples (lanes 1–7, 10% each) and the GST pull-down samples (lanes 8–13) were resolved by SDS-PAGE, and His-tagged eIF2A and GST-fusion proteins were observed by Western blot analyses using anti-His and anti-GST antibodies, respectively.

**Fig. S5** RNA–protein interactions among eIF2A, HCV IRES, and tRNA_i_^Met^. **a** HCV IRES strongly binds to eIF2A. RNA pull-down experiments were performed using the lysates of 293FT cell ectopically expressing eIF2A or eIF2A (K567A). The biotinylated RNAs corresponding to the HCV or EMCV IRES element were synthesized by in vitro transcription in the presence of biotin-UTP (Roche). Cell lysates (3 mg of proteins) were incubated with streptavidin-agarose resin conjugated with either biotinylated HCV RNA (3 μg) or biotinylated EMCV RNA (3 μg) at 4˚C for 1 h in incubation buffer [0.05% NP-40, 40 mM HEPES–KOH (pH 7.5), 100 mM KCl, 1 mM EDTA, 10 mM β-glycerophosphate, 10 mM NaF, 2 mM Na_3_VO_4_, and 1 mM PMSF]. The resin-bound proteins were analyzed by Western blotting. Wild-type eIF2A strongly interacts with the HCV IRES, but K567A mutant of eIF2A does not. The positive control protein eIF3c strongly interacts with the HCV IRES. On the other hand, the EMCV IRES (negative control RNA) interacts with either eIF2A or eIF3c weakly. **b** tRNA_i_^Met^ does not compete with HCV IRES RNA for binding to eIF2A. RNA pull-down experiments were performed as follows: Biotinylated tRNA_i_^Met^ was conjugated with streptavidin-agarose resin (Pierce) in incubation buffer [0.1% NP-40, 10% glycerol, 40 mM HEPES–KOH (pH 7.5), 100 mM KCl, 1 mM EDTA, 10 mM β-glycerophosphate, 10 mM NaF, 2 mM Na_3_VO_4_, and 1 mM PMSF]. Purified His–eIF2A protein and competitor RNAs were pre-incubated in the incubation buffer at 4˚C for 20 min, and then applied to the resin conjugated with biotinylated tRNA_i_^Met^. Samples were incubated at 4˚C for 30 min, and the resin-bound proteins were analyzed by Western blotting. The results indicate that HCV IRES RNA bound to eIF2A does not inhibit the binding of tRNA_i_^Met^ to the protein.

**Fig. S6** Start codon in the HCV IRES plays a key role in the mRNA-dependent loading of tRNA_i_^Met^ onto the 40S ribosomal subunit. The loading of [^32^P]tRNA_i_^Met^ onto the 40S ribosomal subunit was monitored by filter-binding assays [24] performed using radiolabeled tRNAs, 40S ribosomes, various combinations of eIF2A, eIF5B, together with either the wild-type HCV IRES (panel **a**), or a mutant HCV IRES-containing substitution of the start codon from AUG to AAA (panel **b**). The mutant HCV IRES was generated by a site-directed mutagenesis. The amount of components are as follows: 2 pmol of [^32^P]tRNA_i_^Met^, 2.5 pmol of 40S ribosomal subunit, 2 pmol of IRES RNAs, and 2.5 pmol of eIF2A and eIF5B. The equivalent experiments performed independently are marked with an asterisk (*). Experiments were performed three times, and average values are presented in the middle panel, along with a representative autoradiogram. The columns and bars in the bottom panels represent the means and ± standard deviations, respectively.

As expected, the wild-type HCV IRES enhanced the loading tRNA_i_^Met^ to the 40S ribosome about 2.5-fold (compare column 5 with 2 in panel **a**). On the other hand, the addition of the mutant HCV IRES did not enhance the loading tRNA_i_^Met^ to the 40S ribosome (compare column 4 with 2 in panel **b**). Instead, the mutant HCV IRES partially inhibited the loading tRNA_i_^Met^ to the 40S ribosome. This indicates that the codon–anticodon interaction between the HCV IRES and the tRNA_i_^Met^ plays an important role in mRNA-dependent loading of tRNA_i_^Met^ onto the 40S ribosome which is mediated by eIF2A.

In addition, we investigated the start codon mutation on the loading of tRNA_i_^Met^ onto the 40S ribosome which is mediated by eIF5B. As expected, eIF5B facilitated the loading of tRNA_i_^Met^ onto the 40S ribosome in the presence of the wild-type HCV IRES (compare column 4 with 1 in panel **a**). To our surprise, the loading of tRNA_i_^Met^ onto the 40S ribosome, which was mediated by eIF5B, was completely abolished when the mutant HCV IRES was added in the reaction mixture instead of the wild-type HCV IRES (compare column 5 with 1 in panel **b**). Moreover, the synergistic activation of tRNA_i_^Met^ loading onto the 40S ribosome by eIF2A and eIF5B (shown in Fig. 4) was abolished when the mutant HCV IRES was used in the filter-binding assay (compare column 6 with 4 in panel **b**). This indicates that the codon–anticodon interaction between the HCV IRES and the tRNA_i_^Met^ is absolutely needed for the eIF5B-mediated loading of tRNA_i_^Met^ onto the 40S ribosome. On the contrary, eIF2A-mediated loading of tRNA_i_^Met^ onto the 40S ribosome was possible in the absence of HCV IRES (compare column 2 with 1 in Fig. S6b), and that remained in the presence of the mutant HCV IRES (compare column 4 with 1 in panel **b**). The difference of tRNA_i_^Met^-loading capability of these proteins is likely due to the binding affinity of these proteins with tRNA_i_^Met^. That is, eIF2A but not eIF5B strongly interacts with tRNA_i_^Met^.

In conclusion, the codon–anticodon interaction between mRNA and tRNA_i_^Met^ plays a key role in the mRNA-dependent loading of tRNA_i_^Met^ onto the 40S ribosome that is mediated by either eIF2A or eIF5B.

**Fig. S7** Tunicamycin induces phosphorylation of eIF2α. Western blot analyses were performed with the cell lysates used in sucrose density gradient analyses, as shown in Fig. 5. Western blotting was performed with an anti-eIF5B (Proteintech), anti-eIF2A (Proteintech), and anti-phospho-eIF2α (Ser51) (Cell signaling). As expected, the level of phosphorylated eIF2α was dramatically elevated by the tunicamycin treatment.

**Fig. S8** Lysine at the 567^th^ amino acid of eIF2A is required for the mRNA-dependent augmentation of tRNA_i_^Met^ loading onto the 40S ribosome. The loading of [^32^P]tRNA_i_^Met^ onto the 40S ribosomal subunit was monitored by filter-binding assays [24] performed using radiolabeled tRNAs, 40S ribosomes, HCV IRES, together with either the wild-type eIF2A or mutant eIF2A (K567A). The amount of components are as follows: 2 pmol of [^32^P]tRNA_i_^Met^, 2.5 pmol of 40S ribosomal subunit, 2 pmol of HCV IRES, and 2 pmol of purified proteins [eIF2A (WT) or eIF2A (K567A)].

The mutant eIF2A (K567A) strongly facilitated the loading of tRNA_i_^Met^ onto the 40S ribosome similarly to the wild-type eIF2A in the absence of HCV IRES (compare columns 1 to 3 in Figure S8). As expected, the HCV IRES strongly augmented the loading of tRNA_i_^Met^ onto the 40S ribosome that is mediated by the wild-type eIF2A (compare column 4 with 2). On the contrary, the HCV IRES did not augment the loading of tRNA_i_^Met^ onto the 40S ribosome which was mediated by the mutant eIF2A (K567A) (compare column 5 with 3). The results indicate that the interaction between eIF2A and mRNA, which requires the lysine residue at 567 of eIF2A, plays a key role in the mRNA-dependent loading of tRNA_i_^Met^ onto the 40S ribosome. Experiments were performed four times, and average values are presented in the middle panel, along with a representative autoradiogram. The columns and bars in the bottom panel represent the means and ± standard deviations, respectively.

Supplementary material 1 (PDF 6867 kb)

## Abbreviations

IF2: Prokaryotic initiation factor 2
eIFs: Eukaryotic initiation factors
eIF2A: Eukaryotic initiation factor 2A
eIF5B: Eukaryotic initiation factor 5B
Initiator tRNA: Met-tRNA_i_^Met^
HRI: Heme-regulated inhibitor
PKR: Protein kinase double-stranded RNA-dependent
PERK: PKR-like ER kinase
GCN2: General control non-derepressible-2
IRES: Internal ribosome entry site
HCV: Hepatitis C virus
