# Supplementary material for: eIF2A, an initiator tRNA carrier refractory to eIF2α kinases, functions synergistically with eIF5B
Source: Cell Mol Life Sci. 2018 Jul 17;75(23):4287–300. doi: 10.1007/s00018-018-2870-4 (PMC6208778; doi:10.1007/s00018-018-2870-4)

Fig S1

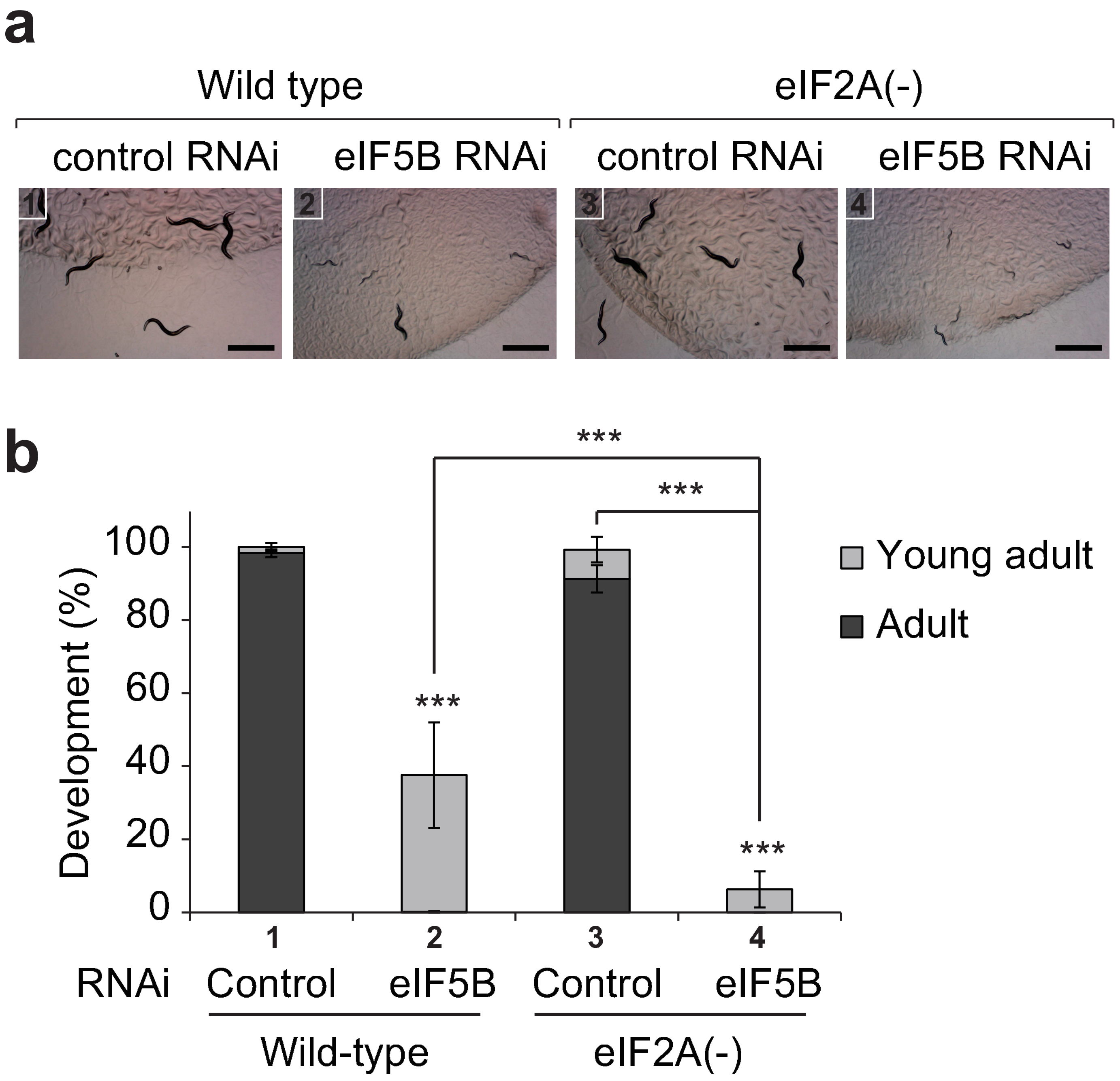
a
Wild type
eIF2A(-)
control RNAi
eIF5B RNAi
control RNAi
eIF5B RNAi
1
2
3
4
b
***
***
***
***
Young adult
Adult
Development (%)
100
80
60
40
20
0
1
2
3
4
RNAi
Control
eIF5B
Control
eIF5B
Wild-type
eIF2A(-)

Fig S2

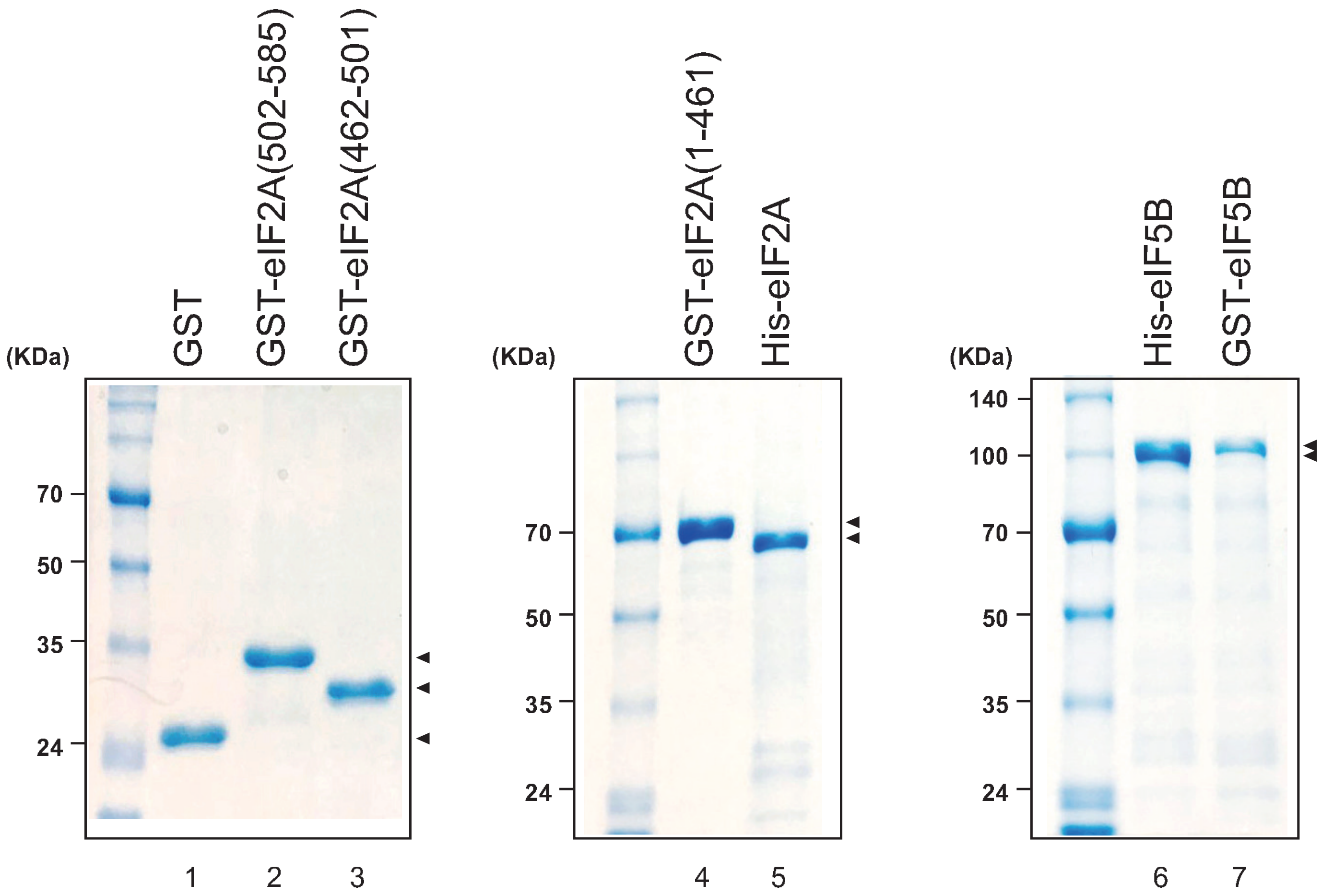

(KDa)
GST
GST-eIF2A(502-585)
GST-eIF2A(462-501)
70
50
35
24
1
2
3
(KDa)
GST-eIF2A(1-461)
His-eIF2A
70
50
35
24
4
5
(KDa)
His-eIF5B
GST-eIF5B
140
100
70
50
35
24
6
7

Fig S3

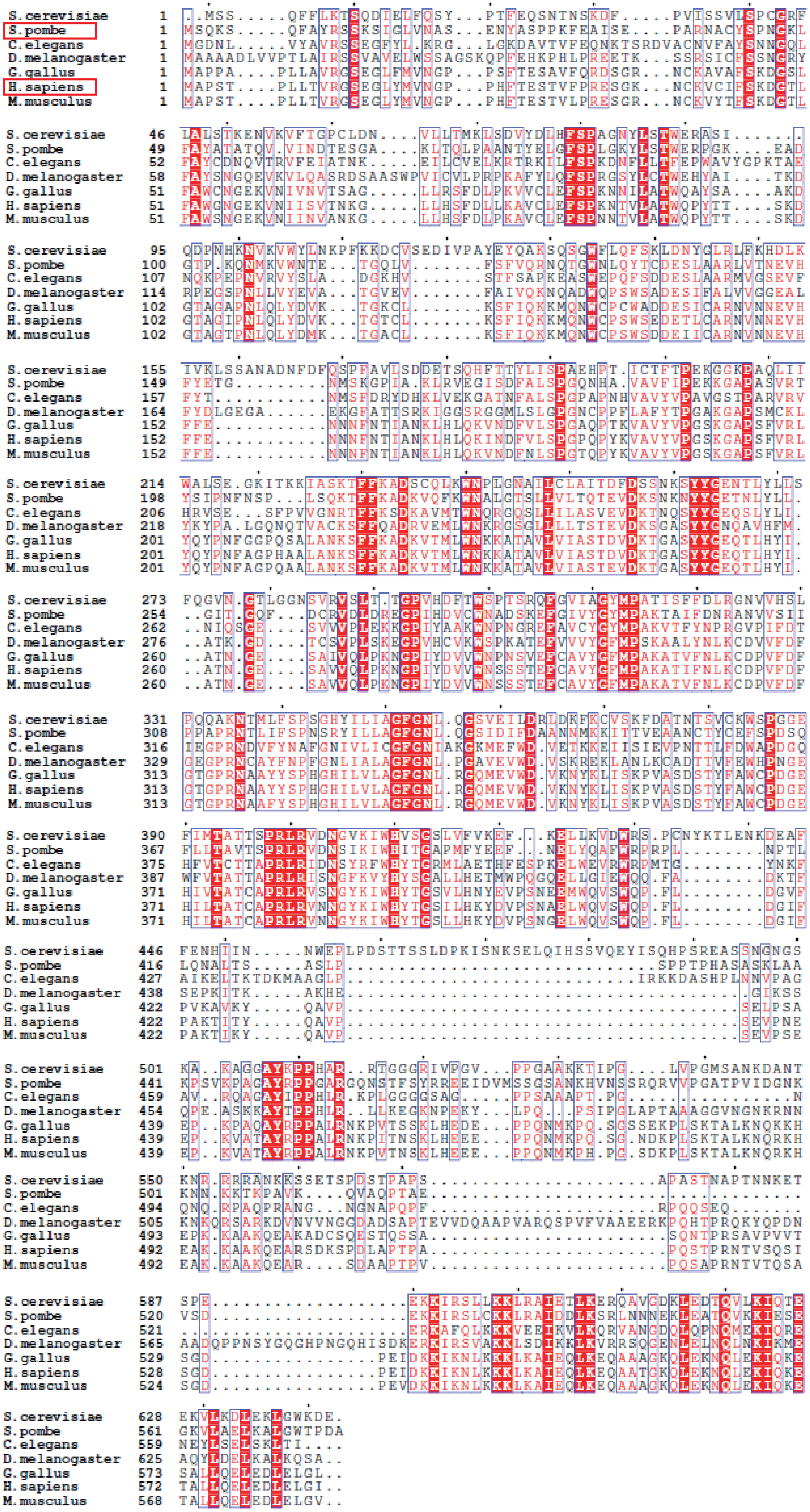
S.cerevisiae
S.pombe
C.elegans
D.melanogaster
G.gallus
H.sapiens
M.musculus

Fig S4

a

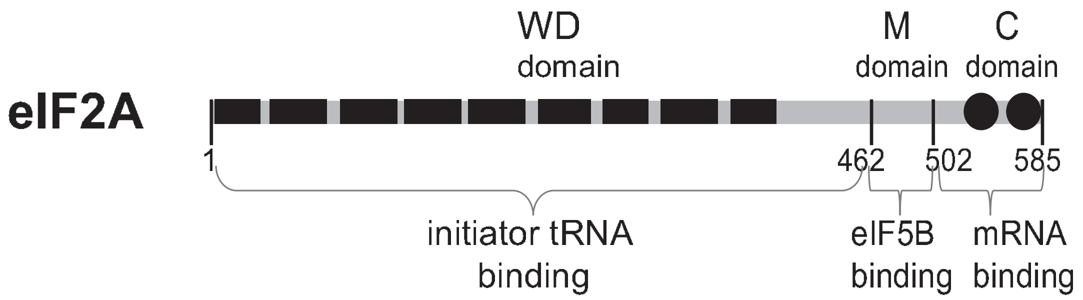
WD
domain
M
domain
C
domain
eIF2A
1
462
502
585
initiator tRNA
binding
eIF5B
binding
mRNA
binding

b

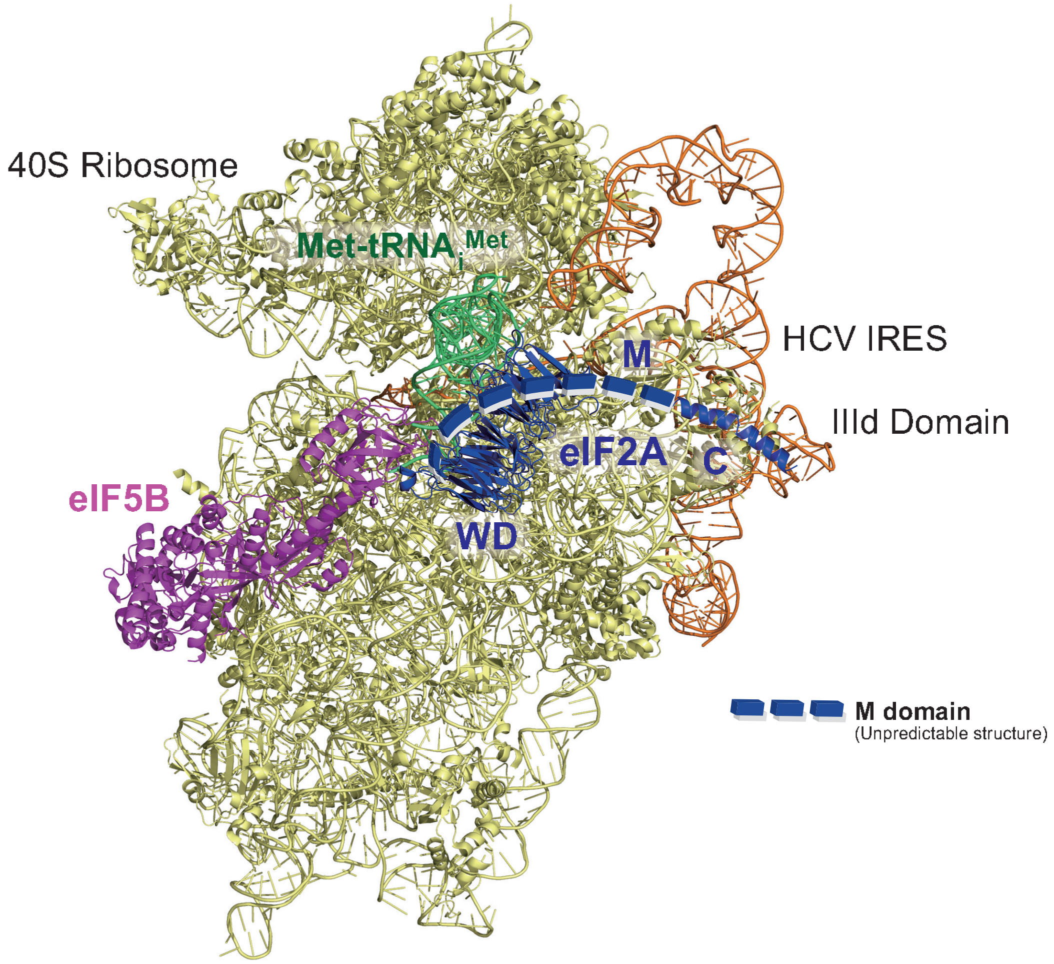
40S Ribosome
Met-tRNAi Met
HCV IRES
M
IIId Domain
eIF2A
C
eIF5B
WD
M domain
(Unpredictable structure)

Fig S4

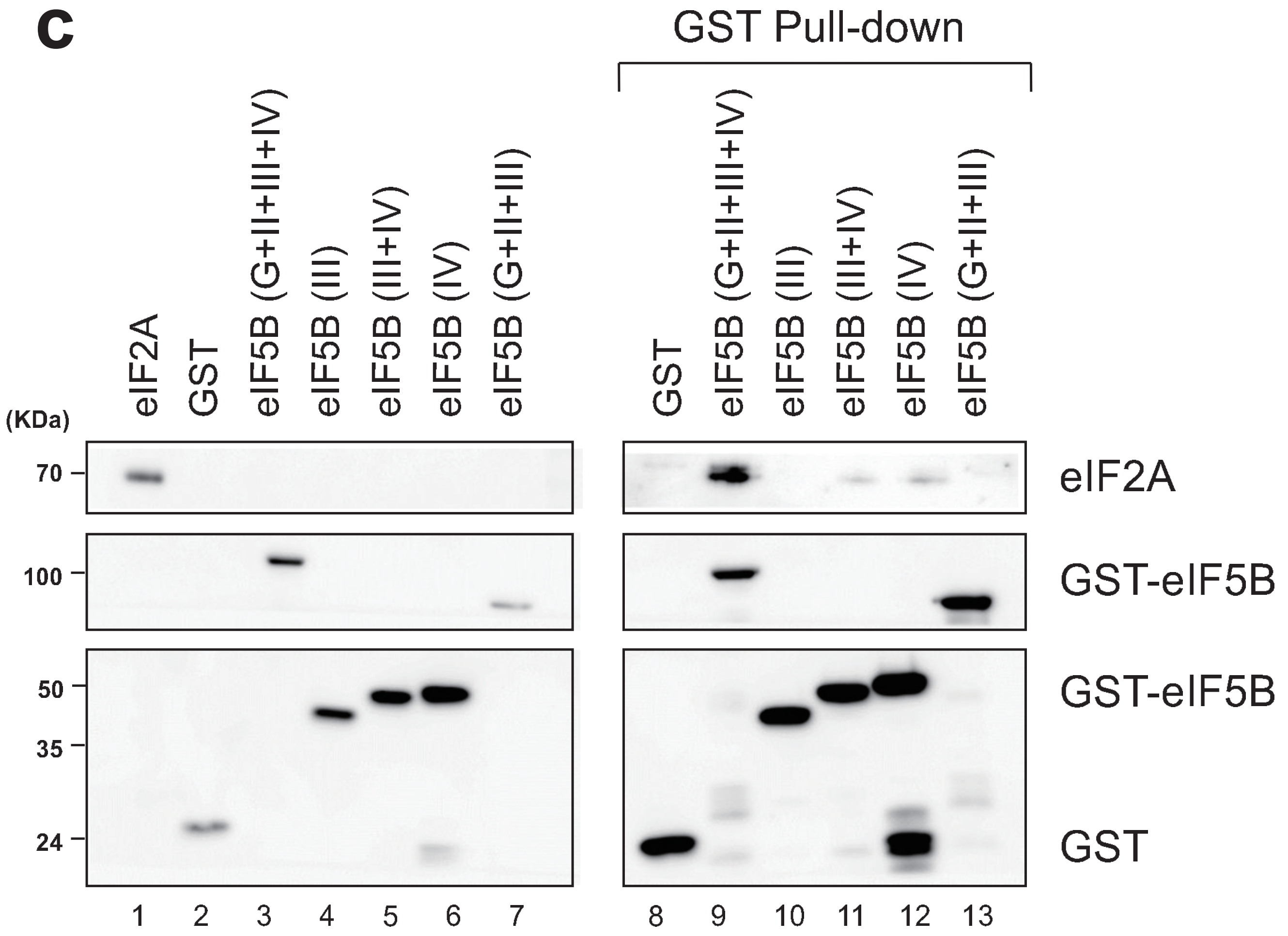
c
GST Pull-down
eIF2A
GST
eIF5B (G+II+III+IV)
eIF5B (III)
eIF5B (III+IV)
eIF5B (IV)
eIF5B (G+II+III)
GST
eIF5B (G+II+III+IV)
eIF5B (III)
eIF5B (III+IV)
eIF5B (IV)
eIF5B (G+II+III)
(KDa)
70
100
50
35
24
eIF2A
GST-eIF5B
GST-eIF5B
GST
1 2 3 4 5 6 7
8 9 10 11 12 13

Fig S5

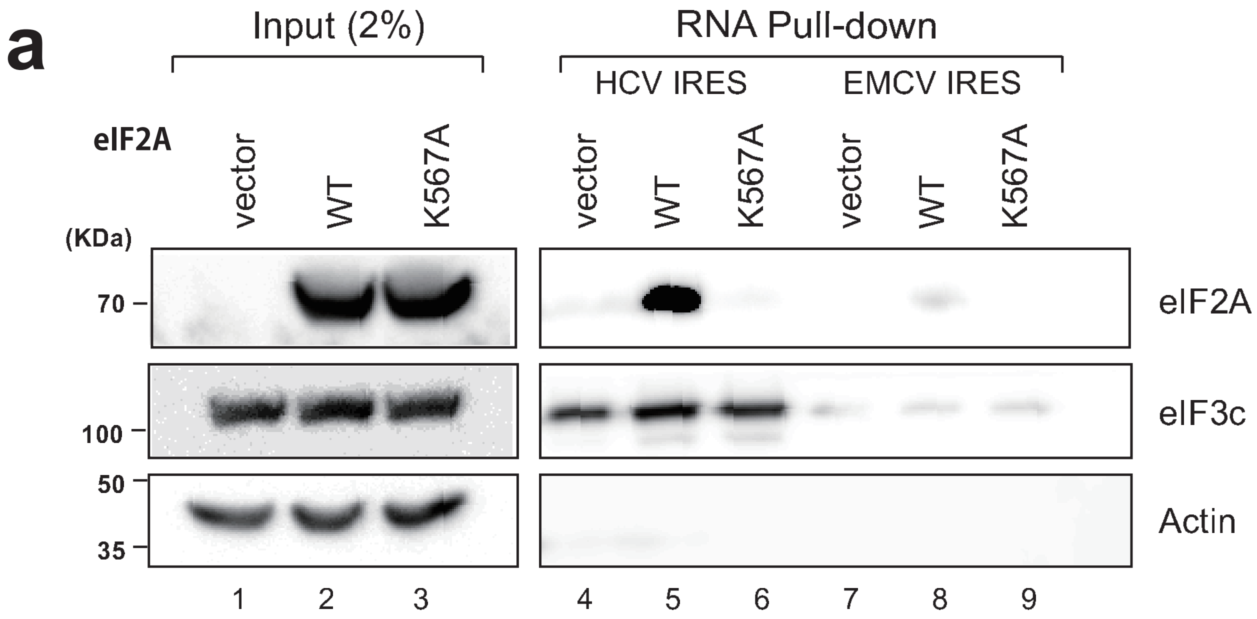
a
Input (2%)
RNA Pull-down
HCV IRES
EMCV IRES
eIF2A
vector
WT
K567A
vector
WT
K567A
vector
WT
K567A
(KDa)
70
100
50
35
eIF2A
eIF3c
Actin
1
2
3
4
5
6
7
8
9

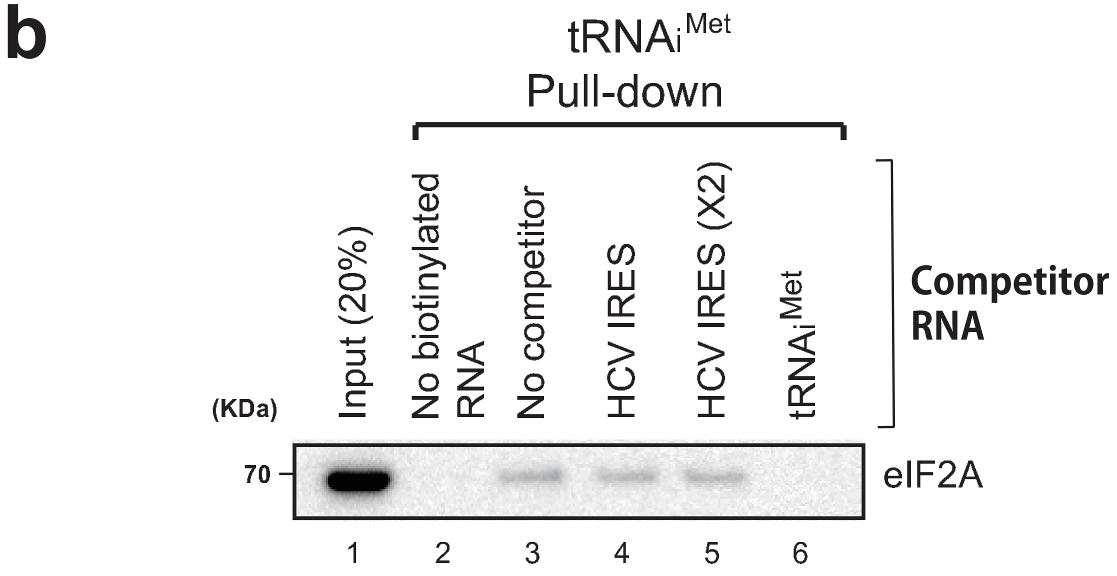
b
tRNAi Met
Pull-down
Input (20%)
No biotinylated RNA
No competitor
HCV IRES
HCV IRES (X2)
tRNAi Met
Competitor RNA
(KDa)
70
eIF2A
1
2
3
4
5
6

Fig S6

a

1	2	3	4	5	
+	+	+	+	+	40S
−	−	+	+	+	HCV IRES (WT)
−	+	−	−	+	eIF2A
−	−	−	+	−	eIF5B
+	+	+	+	+	[^{32}P]tRNA$_i^{Met}$

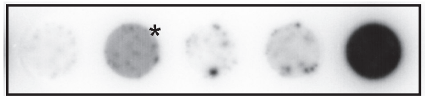

Average value					
	0.07	1.00	0.09	0.66	2.52

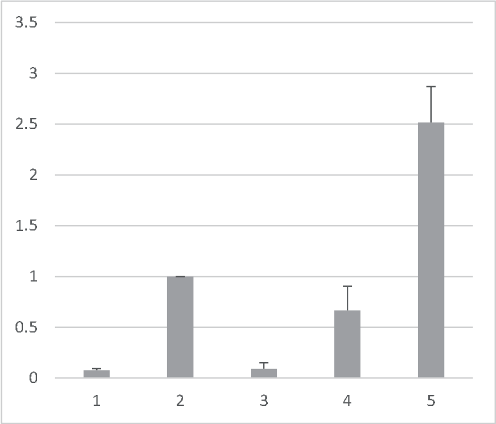

b

1	2	3	4	5	6	
+	+	+	+	+	+	40S
−	−	+	+	+	+	HCV IRES (mt)
−	+	−	+	−	+	eIF2A
−	−	−	−	+	+	eIF5B
+	+	+	+	+	+	[^{32}P]tRNA$_i^{Met}$

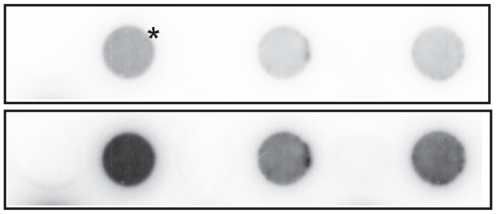

(short exposure)

(Long exposure)

Average value						
	0.06	1.00	0.04	0.54	0.05	0.63

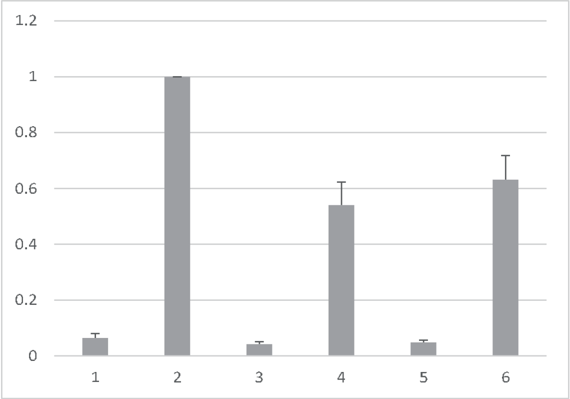

Fig S7

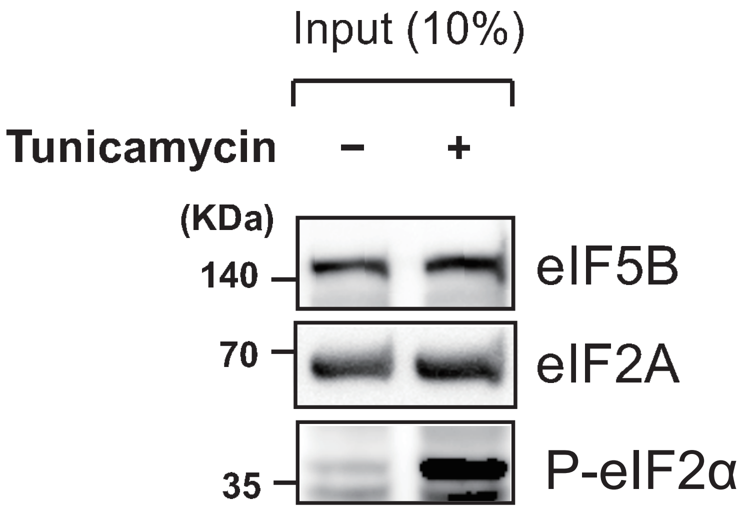

Fig S8

1	2	3	4	5	6	
+	+	+	+	+	−	40S
−	−	−	+	+	+	HCV IRES
−	+	−	+	−	+	eIF2A
−	−	+	−	+	−	eIF2A(K567A)
+	+	+	+	+	+	[^{32}P]tRNA$_i^{Met}$

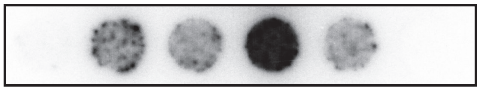

Average value	0.04	1.00	0.71	3.13	0.55	0.14

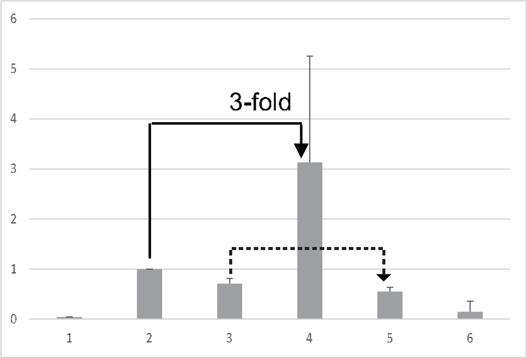

Supplement: Supplementary file 1 — Supplementary material 1 (PDF 6867 kb) [file 18_2018_2870_MOESM1_ESM.pdf]
